# Energy-Efficient Anomaly Detection and Chaoticity in Electric Vehicle Driving Behavior

**DOI:** 10.3390/s24175628

**Published:** 2024-08-30

**Authors:** Efe Savran, Esin Karpat, Fatih Karpat

**Affiliations:** 1 Department of Mechanical Engineering, Bursa Uludag University, 16059 Bursa, Turkey; efesavran@uludag.edu.tr; 2 Electrical-Electronics Engineering Department, Bursa Uludag University, 16059 Bursa, Turkey; esinoz@uludag.edu.tr

**Keywords:** anomaly detection, long short-term memory, local outlier factor, Mahalanobis distance, energy optimization, machine learning, chaoticity

## Abstract

Detection of abnormal situations in mobile systems not only provides predictions about risky situations but also has the potential to increase energy efficiency. In this study, two real-world drives of a battery electric vehicle and unsupervised hybrid anomaly detection approaches were developed. The anomaly detection performances of hybrid models created with the combination of Long Short-Term Memory (LSTM)-Autoencoder, the Local Outlier Factor (LOF), and the Mahalanobis distance were evaluated with the silhouette score, Davies–Bouldin index, and Calinski–Harabasz index, and the potential energy recovery rates were also determined. Two driving datasets were evaluated in terms of chaotic aspects using the Lyapunov exponent, Kolmogorov–Sinai entropy, and fractal dimension metrics. The developed hybrid models are superior to the sub-methods in anomaly detection. Hybrid Model-2 had 2.92% more successful results in anomaly detection compared to Hybrid Model-1. In terms of potential energy saving, Hybrid Model-1 provided 31.26% superiority, while Hybrid Model-2 provided 31.48%. It was also observed that there is a close relationship between anomaly and chaoticity. In the literature where cyber security and visual sources dominate in anomaly detection, a strategy was developed that provides energy efficiency-based anomaly detection and chaotic analysis from data obtained without additional sensor data.

## 1. Introduction

Anomaly detection is the process of identifying deviations from normal behavior in a dataset. These deviations are defined as data points that differ from the normal distribution and often indicate situations such as erroneous data, fraud, security breaches, or unexpected events. Anomaly detection is related to data science and statistics. Methods used in the statistical field assume a normal distribution of the data and define values outside the threshold in this distribution as anomalies. The Z-score and Interquartile Range (IQR) methods, which are the most well-known methods in the studies, are widely used to identify extreme values in data. However, since these methods generally assume the normal distribution of the data, they are limited to complex and multidimensional datasets. Machine learning techniques can meet this deficiency. Various techniques have been developed for anomaly detection [1]. Unsupervised learning algorithms offer a more sophisticated approach to understanding the structure of data and identifying deviations from normal behavior. At the same time, supervised learning algorithms, such as support vector machines (SVMs) and decision trees, are used for anomaly detection and provide successful results [2]. Deep learning techniques, especially models suitable for time series analysis, such as neural networks and LSTM, offer effective solutions for anomaly detection in large datasets and complex time series data. LSTM-Autoencoder has become a widely used method for detecting anomalies by learning normal behavior in time series data. Density-based methods such as the Local Outlier Factor (LOF) also stand out for their ability to detect anomalies in different density regions. Recently, anomaly detection has been moved further with the development of hybrid models. Hybrid models combine the strengths of different methods, providing higher accuracy and reliability [3,4]. In this regard, the combination of LSTM-Autoencoder and methods such as the Mahalanobis distance or LOF has the potential to offer a more robust and comprehensive anomaly detection mechanism by jointly evaluating the dynamic structure of time series data and the distribution of data points.

Anomaly detection is of critical importance in the automotive industry. An anomaly in driving data can be considered an unusual or unexpected behavior that deviates significantly from normal driving patterns. This can be sudden acceleration, irregular braking, erratic steering movements, or any event that does not match normal driving data. Incorrect detection during anomaly detection may cause unnecessary warnings or interventions in the vehicle, causing the driver to worry and question the reliability of the system. In fleets, it may lead to operational inefficiencies and increased costs due to unnecessary inspections or stops. If a serious problem such as aggressive driving or vehicle failure is not detected, high financial losses may occur. For the driver, failure to detect risky behaviors may prevent the improvement in safety and performance. Anomalies can be caused by various factors, such as mechanical problems, sensor failures, driver errors, or potentially dangerous behaviors such as careless or aggressive driving. In addition to physical attacks, software attacks are also carried out on vehicles [5]. The Controller Area Network (CAN) line in vehicles has a dense data flow and provides sufficient data support for anomaly detection. In this way, cyber-attacks can be easily detected with anomaly detection models [6]. Predictive driving can be provided and accident risks can be reduced by monitoring road conditions and detecting abnormal situations [7]. Anomaly detection can also be included in the energy efficiency issue, which has become increasingly important in recent years. Energy management is of critical importance, especially in hybrid and electric vehicles. It can optimize energy efficiency by detecting decreases in battery performance or unexpected increases in energy consumption. With energy optimization, a longer driving range and a happier vehicle user will be possible. Thanks to this system, environmental impacts and financial costs are minimized. Low energy consumption can also reduce resource overconsumption and carbon emissions. A more sustainable transportation system can be achieved.

Collecting real-world driving data is vital in anomaly detection studies. These data are collected from various sensors and electronic control units on the vehicle. The CAN protocol is the main source of such data. CAN data provide information about the speed, engine speed, power, braking, steering angle, and other important parameters. Obtaining driving data can be achieved through real-world driving, in addition to simulations in the laboratory environment. The data collected include variables such as road conditions, weather, and driver behavior, which improves model accuracies by increasing the diversity of the data [8]. The diversity of real-world data brings complexity. Noise and missing data problems have the potential to negatively affect anomaly detection studies [9]. Various strategies have been developed to eliminate these difficulties. Reducing noise and filling in missing data can be achieved through data preprocessing steps. Comprehensive datasets created with data collected from different driving, road, or weather conditions enable models to learn different scenarios. This process ensures that new anomaly types and changing driving conditions can be transferred to the anomaly detection model. Although the use of real-world data makes anomaly detection studies more challenging, the richness and diversity offered by these data allow the development of more reliable and effective anomaly detection systems.

Combining well-known methods with strong aspects under appropriate conditions provides a more effective and comprehensive approach. In the literature [10,11,12], approaches implemented with hybrid models have provided very successful results. LSTM-Autoencoder is used to detect anomalies in time series data [13,14]. It is flexible enough to handle incomplete and noisy data. This model tries to minimize the reconstruction error by encoding the data into a low-dimensional space. Data with high reconstruction errors are considered potential anomalies. LSTM-Autoencoder is especially effective at learning complex relationships in time series data. It is used in natural language processing, speech recognition, and video analysis. The LOF is a local-density-based anomaly detection algorithm [15,16]. It calculates the anomaly score by comparing the local density of the data point with the surrounding points. Points located in low-density regions have a high anomaly score and are therefore labeled as anomalies. The LOF is highly effective at detecting local anomalies in datasets. The Mahalanobis distance measures how far a data point is from a distribution [17,18,19]. This distance index detects multidimensional anomalies by taking into account the covariance structure of data points. It is especially effective at detecting anomalies in high-dimensional data. Hybrid models created by combining these methods provide many benefits in anomaly detection. LSTM-Autoencoder learns complex relationships in time series data, while the LOF detects local density differences and the Mahalanobis distance identifies high-dimensional anomalies. This combination improves the overall model performance by detecting different types of anomalies more effectively. Hybrid models provide strong performances in different scenarios because each method has the potential to capture different types of anomalies with its strengths.

Chaoticity assessment in vehicle driving data plays a critical role in developing vehicle technologies and improving driving safety. Similar to its effects in other numerical studies [20], chaotic analysis provides a better understanding of driving behavior by revealing the complexity and unpredictability of driving dynamics. In this way, it makes a significant contribution to anomaly detection studies in driving data. This type of assessment analyzes how the driver copes with sudden maneuvers, speed changes, and unexpected situations and helps predict dangerous situations that may arise while driving. Additionally, chaotic features in driving data provide important information that can be used to optimize the driving performance and increase energy efficiency. For example, the chaotic nature of instantaneous speed changes or sudden changes in road conditions while driving can be optimized to enable driver assistance systems to respond faster and more accurately. This improves overall road safety and helps prevent accidents. Performing chaotic analysis along with anomaly detection expands the scope of the study and deepens the dataset analysis.

There are many previous studies on this subject. When the studies most similar to this study are examined, in the study of Qin [5] and others, anomaly detection was made with LSTM support over CAN bus data, and loss function performance comparisons were made in five different forms. In the study, the loss function with the best performance was determined. Moso et al. [21] conducted an anomaly detection study using vehicle data streams to describe road anomalies. The Harvesine distance metric and ball tree k-nearest neighbor (KNN) management were used in the study. The developed technique showed a superior performance in anomaly detection. Yun et al. [22] developed a machine learning-based detection system for driving anomalies in autonomous vehicles caused by connectivity issues, sensor failure, misorientation, or environmental impacts. Thanks to the units placed on the roadside, abnormal driving behavior of the vehicles was detected. Three different scenarios were simulated in the study. The ability of machine learning algorithms to detect these anomalies has been evaluated. The Minisom algorithm stands out with its superior performance. Wickramasinghe et al. [6] present an anomaly detection system for anomaly detection in the CAN protocol communication of electric vehicles. The developed system offers a window-based feature engineering approach that extracts cyber features from the internal communication data of electric vehicles. The ResNet Autoencoder, adversarial machine learning, LOF, and One-Class SVM (OCSVM) methods were used in the study. Additionally, by combining the Autoencoder and adversarial machine learning approaches, it enabled both anomaly detection and an explanation of the detected anomalies. It has been shown that the developed model can provide an effective and reliable solution for anomaly detection and annotation generation in the CAN data communication of electric vehicles. The study by Barbosa et al. [23] includes the evaluation of the structural integrity of the Lyapunov exponent. Physical nonlinearities were considered for the structure. It was observed that the Lyapunov exponent is a usable parameter to evaluate the structural integrity and is effective for detecting dynamic changes and material problems. Vogl [24] examined whether measures of chaos in financial markets change over time and analyzed the usability of chaos measurements in predicting financial markets and whether these measurements vary over time. For chaotic measurements, the Hurst Exponent, Maximum Lyapunov Exponent, and Sample Entropy were calculated. Entropy increases have been observed during crisis periods, indicating that the self-similarity of the system decreases and chaos increases. Significant changes during crisis periods suggest the usability of measurements in crisis predictions. Tian [25] analyzed the chaotic characteristics of network traffic time series at different time scales. Power Spectral Density and Autocorrelation analyses were performed to qualitatively explain the chaotic behavior. Phase space reconstruction was performed to calculate the largest Lyapunov exponent and Kolmogorov entropy. The largest Lyapunov exponent and Kolmogorov entropy in the results of the study showed the chaotic nature of the network traffic and the rate of information loss over time. As the time scale increased, the Kolmogorov entropy also increased, indicating that the chaotic properties became stronger, and the predictability decreased.

Numerous studies in the literature have made significant contributions to this field. However, there are still some deficiencies in anomaly detection and chaoticity analysis. The studies included in the literature do not have an energy efficiency perspective in anomaly detection. Road quality has mostly been evaluated to ensure driving safety. Similarly, CAN bus data have been evaluated from a cyber-security perspective. Chaoticity analysis has been considered independently. However, the sustainability perspective should also be included in the prediction of risky situations in mobility. In this study, two different hybrid models were developed using the combination of the LSTM-Autoencoder, LOF, and Mahalanobis distance methods, which are frequently mentioned in the literature, and the anomaly detection performance was improved compared to that of traditional methods. Anomaly detection was performed on two different real-world driving datasets. It was seen that the methods developed in this study, which also included energy consumption analysis, could successfully perform anomaly analysis on CAN data. In addition, the chaotic nature of the datasets was also taken into account with the help of appropriate metrics.

The specific contributions of this study to the existing literature are outlined as follows:

- Generalization of the models was achieved by using multiple real-world driving data;
- The performance of the existing anomaly detection techniques has been increased with unsupervised hybrid anomaly detection models;
- Excessive energy consumption was revealed with the help of anomaly detection, contributing to energy efficiency in electric vehicles;
- The dataset was also evaluated with chaoticity metrics, and the future prediction of driving behaviors was also supported.

## 2. Materials and Methods

**Figure 1:**
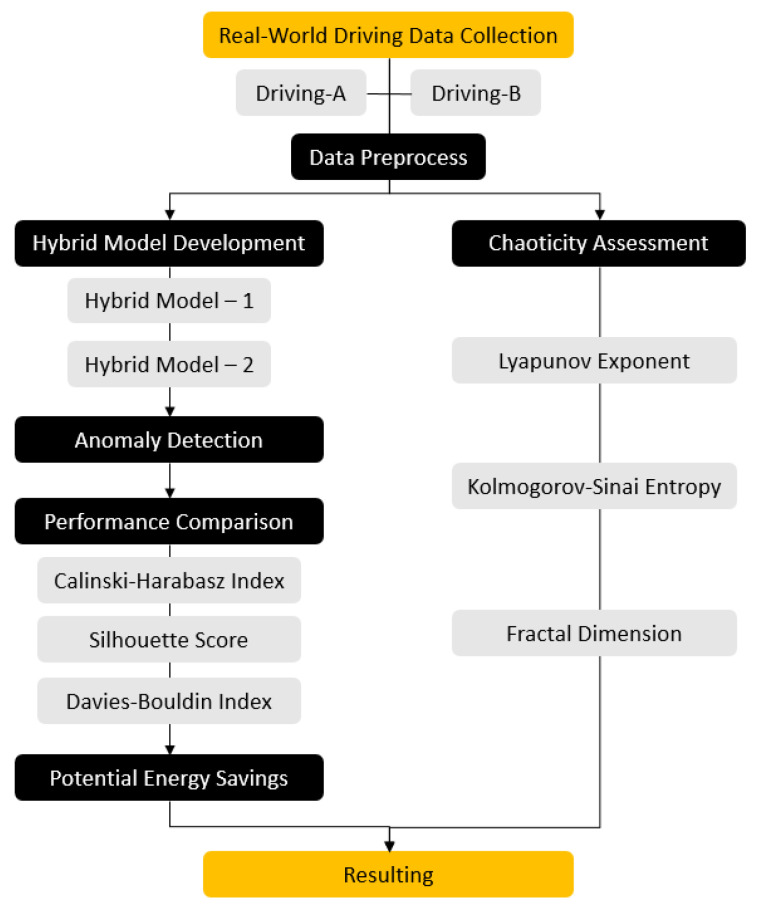
Process flow of the study.

Two hybrid models were developed for unsupervised anomaly detection based on real-world driving data. To evaluate the performances of the models, an electric vehicle was driven twice under the same conditions and the CAN bus data were recorded. CAN records were preprocessed to make them suitable for hybrid models. Hybrid models were created with the LSTM-Autoencoder, LOF, and Mahalanobis distance sub-methods. The silhouette score, Davies–Bouldin index, and Calinski–Harabasz index were calculated for the anomaly detection performance evaluation. Excessive energy consumption caused by detected anomalies was calculated. In parallel, the Lyapunov exponent, Kolmogorov–Sinai entropy, and fractal dimension were calculated to evaluate the chaoticity of the driving data. At the end of this flow, it was revealed that there was a relationship between anomaly and chaoticity, and the anomaly detection outputs were strengthened. The flow of the study is seen in Figure 1.

### 2.1. Real-World Driving Data

**Figure 2:**
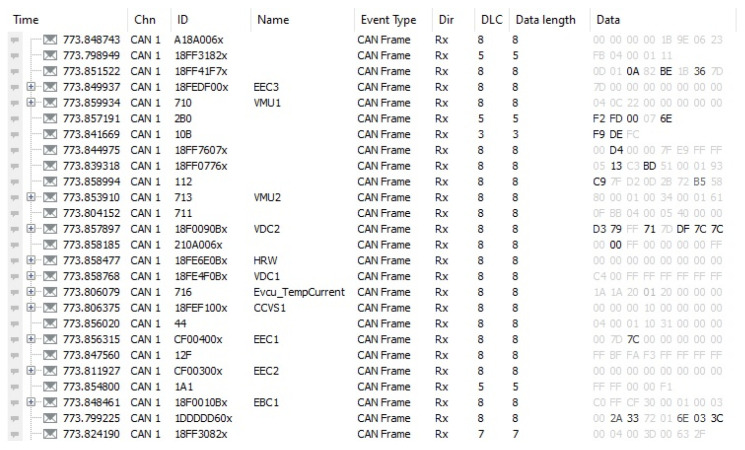
A scope of CAN bus record.

Two different real-driving datasets were used to develop the anomaly detection models. The datasets were obtained as a result of two different people driving a battery electric vehicle suitable for urban use under the same environmental conditions. The hexadecimal-based CAN bus data transferred via the vehicle’s OBD-II port while driving were converted to a CSV file via the VECTOR tool. The preprocessing process for the anomaly detection model was started by extracting vehicle speed, acceleration, throttle ratio, motor torque, and motor power data from the data obtained with a 0.01 s step interval. A scope from the CAN recording while driving can be seen in Figure 2.

**Figure 3:**
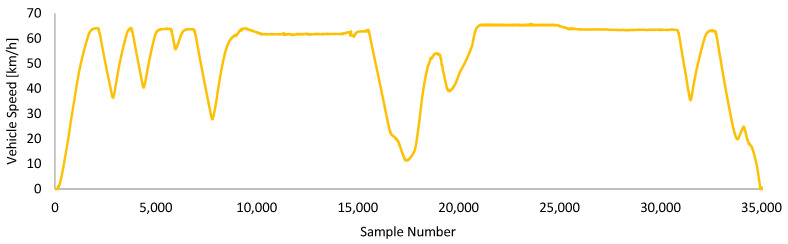
Speed graph of Driving-A.

Figure 3 is the speed graph of the first drive called “Driving-A”. Instead of creating a crowd by sharing the graph of the entire recorded dataset, only the speed graph is shared to represent the driving. These drive data have 35,000 points and include semi-city and semi-highway driving. The vehicle started to move from rest. There was no wind in the driving environment and the ambient temperature was approximately 15 °C. The road occasionally contained slight potholes and bumps. The total energy consumption for this drive was measured as 1.7055 kWh.

**Figure 4:**
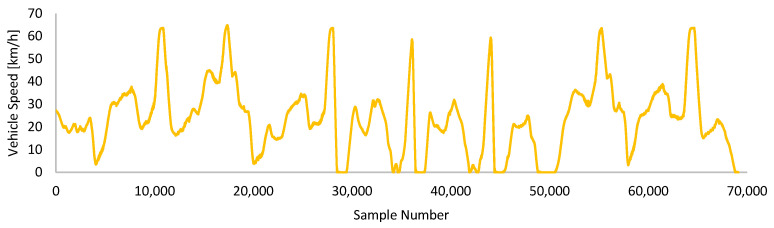
Speed graph of Driving-B.

Figure 4 is the speed graph of the second drive called “Driving-B”. For this driving dataset, only the speed graph is shared to represent the driving. This drive has 70,000 points and represents mostly urban driving. Frequent stop-start and harsh braking are the majority. Driving-B was made on the same route as Driving-A and was in the same environmental conditions. During this drive, the vehicle consumed 0.1129 kWh of total energy.

**Figure 5:**
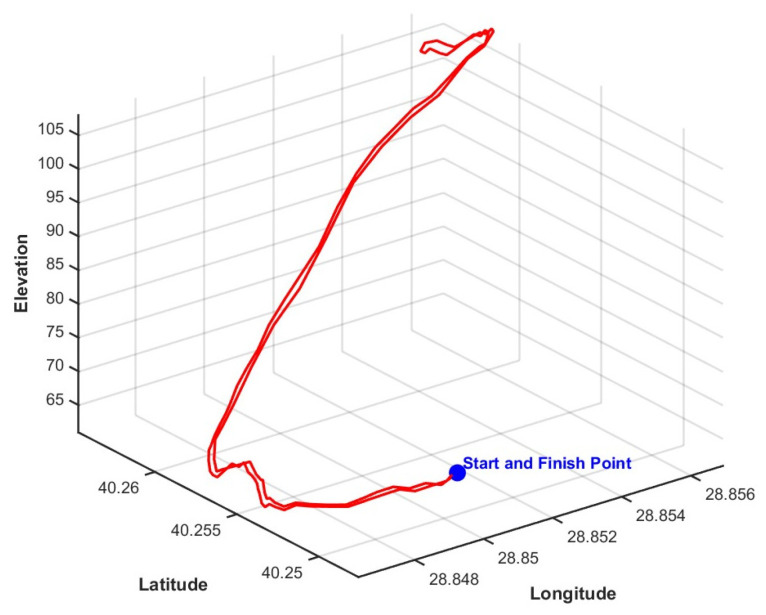
Three-dimensional plot of the real driving route.

Figure 5 provides a three-dimensional visual of the route the battery electric vehicle was driven during the data collection process. The visual was obtained by creating the driving route on a browser-based public platform [26], obtaining the .GPX file, and converting it to the appropriate format in MATLAB R2022b.

### 2.2. Data Preprocessing

It is important to use correct data to achieve correct results in machine learning and statistical numerical processing processes. For this reason, there are many data preprocessing techniques in the literature [27,28,29]. Thanks to data preprocessing techniques, raw data received from sensors are prepared for processing by going through a series of processes. In this study, data preprocessing techniques in the literature were used to obtain accurate results from the anomaly detection processes. Outliers in the data can cause excessive abnormal deviations during the training of the model and produce incorrect results. Outliers in the data were detected with the help of the Z-score criterion, marked as “NaN”, cleaned, and filled with appropriate values using the linear interpolation method with the “fillmissing” function, one of the built-in functions of MATLAB. Data at different scales can reduce the model performance. Normalization allows the model to perform better by pulling the dataset to a common scale. Again, the data were normalized with the help of the “normalize” function in MATLAB. Noise in the data can also prevent the model from producing accurate results. Reducing noise provides a cleaner and more reliable dataset. The noise was reduced using the “movemean” function in MATLAB.

### 2.3. Anomaly Detection Mechanisms

In the context of driving data, anomaly detection aims to identify deviations from typical driving patterns, which can be categorized into three primary classes: driver-based, road-based, and vehicle-based anomalies. Driver-based anomalies encompass behaviors such as inappropriate speed variations relative to traffic or road conditions, sharp turns, sudden braking, and inconsistent accelerator pedal usage. Road-based anomalies include abrupt changes in vehicle acceleration, suspension dynamics, speed data, motor torque, energy consumption, and discrepancies in wheel speeds. Vehicle-based anomalies involve issues such as inconsistencies between the pedal position and motor torque, irregular energy consumption patterns, inadequate deceleration during braking, and erroneous sensor data transmission. Understanding and detecting these anomalies is crucial for anticipating potential risks and developing preventive systems.

Two hybrid models were developed by integrating LSTM-Autoencoder with the LOF and Mahalanobis distance methods. In both models, anomalies detected by the sub-methods were combined to enhance the detection accuracy. The aim was to achieve at least a 5% improvement in the anomaly detection performance by addressing the limitations of the sub-methods through the hybrid models. This approach leverages the strengths of deep learning-based autoencoders alongside statistical and outlier detection methods, resulting in a more flexible and robust model. By combining the anomaly outputs rather than mixing algorithms or features, the model achieves higher interpretability and expands the anomaly detection coverage. The hybrid structure’s flexibility makes the model applicable to various real-world scenarios, particularly in dynamic, time series, and multidimensional anomaly detection, where it is expected to yield successful results.

Hybrid Model-1 includes the LSTM-Autoencoder and LOF methods. The anomaly detection results of the two methods were superposed, and the silhouette score, Davies–Bouldin index, and Calinski–Harabasz techniques were used for the performance evaluation. Hybrid Model-2 detects anomalies using the LSTM-Autoencoder and Mahalanobis distance methods. The operating system and performance evaluation are the same as those of the first model. The pseudocodes of Hybrid Model-1 and Hybrid Model-2 are shared in Algorithms 1 and 2, respectively.

LSTM-Autoencoder is a deep learning model used to learn patterns in time series data [13,14]. Autoencoders attempt to restore the input to its original state by compressing the input (called an encoder) and restructuring it (called a decoder). While restructuring the data in this process, it calculates the differences (errors) between the original data and the reconstructed data. These errors are called reconstruction errors. Reconstruction errors tend to be low for normal data because the model has learned the typical patterns of that data well. In abnormal data, the reconstruction errors are higher because there are differences that the model does not or cannot learn. These high errors are used for anomaly detection. The encoder compresses the input data to create a lower-dimensional representation. LSTM cells process consecutive data of time series data and transfer these data to a hidden state at each step. This latent state captures the dynamics of the data over time. The hidden states created by the encoder create a low-dimensional representation of the data. This representation contains the most important features of the original data and can be thought of as a compressed dataset. Working from this low-dimensional representation, the decoder tries to reconstruct the original input. LSTM cells reconstruct the time series using hidden states. In this process, an attempt is made to create the data closest to their original form by using the important features extracted by the encoder. The decoder layer returns from hidden states to the original data size and reconstructs the data. This method is used in time series prediction, natural language processing, speech recognition, and video analysis.

In the LSTM-Autoencoder model, the hyperparameters were carefully selected to balance the learning efficiency and model complexity. The decision to use 64 neurons within the LSTM layers was made to provide a sufficient level of complexity, ensuring the model could capture essential patterns in the data without leading to overfitting or excessively long training times. Although a larger number of neurons could enhance the model’s capacity, it also risks diminishing returns by increasing the potential for overfitting. The number of epochs was set to 200, which allowed the model to learn effectively from the training data. This choice was supported by the root mean square error (RMSE) graph, which showed stability well before reaching 200 epochs, indicating that this number of iterations was adequate for preventing overtraining. A batch size of 64 was chosen to balance the memory usage with the training efficiency, allowing for effective weight updates while managing the memory consumption and promoting a stable learning process. A dropout rate of 20% was applied, where 20% of the neurons were randomly disabled during each training step to prevent overfitting, a technique widely supported by the literature for its effectiveness in similar contexts. The initial learning rate was set at 0.01, a common choice that balances the trade-off between the convergence speed and model accuracy. Higher learning rates might hinder the model from reaching an optimal solution, while lower rates could unnecessarily prolong the training process. The Adam (Adaptive Moment Estimation) optimization algorithm was selected for its robust performance across various deep learning applications. Additionally, gradient clipping was applied with a threshold of 1 to maintain stability during the learning process, preventing any gradient from exceeding this magnitude and thus avoiding instability. A validation frequency of 10 was set to ensure that the model was tested against the validation set every 10 steps, allowing for the timely monitoring of the validation performance and the early detection of overfitting. The combination of these hyperparameters facilitated a well-balanced learning process, enabling the model to achieve satisfactory accuracy within a reasonable training duration.

To see the performance of LSTM-Autoencoder, one of the anomaly detection methods used in the study, the root mean square error (RMSE) and reconstruction errors of the learning process were examined. The formulas of the specified performance evaluation techniques are expressed in Equations (1) and (2). Equation (1) expresses the calculation of the RMSE value, which is one of the performance evaluation techniques of the LSTM training process. In Equation (1), n is the number of data, r_i_ is the real value, and pi is the predicted value:

(1) $$RMSE=\sqrt{\frac{1}{n}\sum_{i=1}^{n}{\left(r_{i}-p_{i}\right)}^{2}}$$

Equation (8) expresses the calculation of the configuration error used in the performance evaluation of the LSTM-Autoencoder model. In Equation (8), O represents the real data, and R represents the reconstructed value:

(2) Reconstruction Error=|O−R|

The LOF is one of the density-based anomaly detection algorithms, and it identifies anomalies by comparing each data point with its surrounding local density [15,16]. Data points whose density is lower than their neighbors are considered abnormal. This method is especially effective on datasets that vary in density. The LOF is used in processes such as anomaly detection, fraud detection, data cleaning, cyber-attacks, and security analysis. In the LOF anomaly detection sub-model, the number of neighbors was selected as 20, the distance metric was Euclidean, and the anomaly LOF score threshold was selected as 1. The formulas for the calculations used in the LOF method are given in Equations (3)–(5).

Equation (9) shows the reachability distance (RD) between points. In Equation (9), the x and y points are the distances that will make k x take its maximum value. The “dist” enables the calculation of the Euclidean distance between the x and y points.

(3) $$RD\left(x,y\right)=max\{k-distance\left(y\right),dist\left(x,y\right)\}$$

Equation (4) is the formula for the local reach density (LRD) of a data point, which is the inverse of the average reach distance of x’s neighbors. In Equation (10), N refers to the neighbor numbers of x:

(4) $$LRD\left(x\right)=\frac{N_{x}}{\sum RD(x,y)}$$

Equation (5) is the formula for the local anomaly factor of a data point. It is calculated by comparing the local access density of x with the local access density of its neighbors:

(5) $$LOF\left(x\right)=\frac{\sum \frac{{LRD}_{y}}{{LRD}_{x}}}{N_{x}}$$

LOF values are generally close to 1, indicating that the data point is normal. As the LOF value increases, the probability that the data point is abnormal increases. In particular, LOF values greater than 1 may indicate that the data point is an anomaly.

The Mahalanobis distance [17,18,19] is a statistical measure used to measure how far a data point is from the rest of the dataset. This distance can be thought of as the Euclidean distance normalized by the covariance matrix of the data points. It provides a more accurate measure of distance by taking into account related variables and the distribution of data points in the dataset. It is especially used for high-dimensional data and in cases where the relationships between the variables are important. In detecting anomalies with the Mahalanobis distance, the covariance matrix and mean vector representing the relationships between the variables are calculated for the normalized dataset. After this stage, the Mahalanobis distance (MD) is calculated for each data point with the formulation given in Equation (6):

(6) $$MD=\sqrt{\frac{{\left(x-\varphi \right)}^{T}}{\sum \left(x-\varphi \right)}}$$

In Equation (6), x is the data point; φ is the average value for each feature; Σ is the covariance matrix containing the variances and covariances of the features.

After determining the Mahalanobis distances, the anomaly detection threshold is determined. This threshold is determined by adding one standard deviation to the mean Mahalanobis distance. Mahalanobis distances exceeding the threshold are labeled as anomalous. The overall anomaly rate and anomaly rates for each feature are calculated. These ratios are used to evaluate the performance of the anomaly detection model.
**Algorithm 1** Hybrid Model-1**Input:** Original data, LSTM-Autoencoder anomaly labels, LOF anomaly labels **Output:** Anomaly rates per feature, overall anomaly rate, evaluation scores, potential energy savings 1: **Load** LSTM-Autoencoder anomaly labels 2: **Load** LOF anomaly labels 3: Read and normalize original data 4: Create hybrid anomaly labels (LSTM-Autoencoder AND LOF) 5: **for** each feature i in features do 6:    labels <- combine LSTM-Autoencoder and LOF anomaly matrices for feature i 7:    silhouetteScores <- calculateSilhouette(normalizedFeatures[i], labels) 8:    meanSilhouetteScore <- mean(silhouetteScores) 9:     dbi <- calculateDaviesBouldin(normalizedFeatures[i], labels) 10:    chs <- calculateCalinskiHarabasz(normalizedFeatures[i], labels) 11:    **Print** meanSilhouetteScore, dbi, chs, anomaly rate for feature i 12: **end for** 13: overallAnomalyRate <- calculateOverallAnomalyRate(hybridLabels) 14: **Print** overallAnomalyRate 15: anomalousPower <- identifyAnomalousPower(power, hybridLabels) 16: potentialEnergySavings <- calculateEnergySavings(anomalousPower) 17: **Print** potentialEnergySavings 18: **for** each feature i in features do 19:    **Plot** normal and anomalous data points for feature i 20: **end for** **function** calculateDaviesBouldin(X, labels) k <- max(labels) clusterMeans <- zeros(k, size(X, 2)) clusterS <- zeros(k, 1) **for** i <- 1 to k do clusterPoints <- X[labels == i, :] clusterMeans[i, :] <- mean(clusterPoints, 1) clusterS[i] <- mean(sqrt(sum((clusterPoints - lusterMeans[i, :]).^2, 2))) **end for** R <- zeros(k) **for** i <- 1 to k do **for** j <- 1 to k do **if** i != j then R[i, j] <- (clusterS[i] + clusterS[j])/sqrt(sum((clusterMeans[i, :] − clusterMeans[j, :]).^2)) **end if** **end for** **end for** D <- max(R, [], 2) dbi <- mean(D) **return** dbi **end function** **function** calculateCalinskiHarabasz(X, labels) k <- max(labels) n <- size(X, 1) clusterMeans <- zeros(k, size(X, 2)) overallMean <- mean(X) betweenClusterDispersion <- 0 withinClusterDispersion <- 0 **for** i <- 1 to k do clusterPoints <- X[labels == i, :] clusterSize <- size(clusterPoints, 1) clusterMeans[i, :] <- mean(clusterPoints, 1) betweenClusterDispersion <- betweenClusterDispersion + clusterSize × sum((clusterMeans[i, :] − overallMean).^2) withinClusterDispersion <- withinClusterDispersion + sum(sum((clusterPoints − clusterMeans[i, :]).^2)) **end for** chs <- (betweenClusterDispersion/(k − 1))/(withinClusterDispersion/(n − k)) **return** chs **end function**

**Algorithm 2** Hybrid Model-2**Input:** Original data, LSTM-Autoencoder anomaly labels, Mahalanobis anomaly labels **Output:** Anomaly rates per feature, overall anomaly rate, evaluation scores, potential energy savings 1: **Load** LSTM-Autoencoder anomaly labels 2: **Load** Mahalanobis anomaly labels 3: Read and normalize original data 4: Create hybrid anomaly labels (LSTM-Autoencoder OR Mahalanobis) 5: **for** each feature i in features do 6:    labels <- combine LSTM-Autoencoder and Mahalanobis anomaly matrices for feature i 7:    silhouetteScores <- calculateSilhouette(normalizedFeatures[i], labels) 8:    meanSilhouetteScore <- mean(silhouetteScores) 9:    dbi <- calculateDaviesBouldin(normalizedFeatures[i], labels) 10:    chs <- calculateCalinskiHarabasz(normalizedFeatures[i], labels) 11:    **Print** meanSilhouetteScore, dbi, chs, anomaly rate for feature i 12: **end for** 13: overallAnomalyRate <- calculateOverallAnomalyRate(hybridLabels) 14: **Print** overallAnomalyRate 15: anomalousPower <- identifyAnomalousPower(power, hybridLabels) 16: potentialEnergySavings <- calculateEnergySavings(anomalousPower) 17: **Print** potentialEnergySavings 18: **for** each feature i in features do 19:    **Plot** normal and anomalous data points for feature i 20: **end for** **function** calculateDaviesBouldin(X, labels) k <- max(labels) clusterMeans <- zeros(k, size(X, 2)) clusterS <- zeros(k, 1) **for** i <- 1 to k do clusterPoints <- X[labels == i, :] clusterMeans[i, :] <- mean(clusterPoints, 1) clusterS[i] <- mean(sqrt(sum((clusterPoints − clusterMeans[i, :]).^2, 2))) **end for** R <- zeros(k) **for** i <- 1 to k do **for** j <- 1 to k do **if** i != j then R[i, j] <- (clusterS[i] + clusterS[j])/sqrt(sum((clusterMeans[i, :] − clusterMeans[j, :]).^2)) **end if** **end for** **end for** D <- max(R, [], 2) dbi <- mean(D) **return** dbi **end function** **function** calculateCalinskiHarabasz(X, labels) k <- max(labels) n <- size(X, 1) clusterMeans <- zeros(k, size(X, 2)) overallMean <- mean(X) betweenClusterDispersion <- 0 withinClusterDispersion <- 0 **for** i <- 1 to k do clusterPoints <- X[labels == i, :] clusterSize <- size(clusterPoints, 1) clusterMeans[i, :] <- mean(clusterPoints, 1) betweenClusterDispersion <- betweenClusterDispersion + clusterSize × sum((clusterMeans[i, :] − overallMean).^2) withinClusterDispersion <- withinClusterDispersion + sum(sum((clusterPoints − clusterMeans[i, :]).^2)) **end for** chs <- (betweenClusterDispersion/(k − 1))/(withinClusterDispersion/(n − k)) **return** chs **end function**

Time complexity analysis was performed to estimate the time spent by the hybrid models developed for anomaly detection. Time complexity expresses how the algorithm scales with the input size using Big-O notation [30]. This analysis, which is performed to understand how efficient an algorithm is, provides insight into the running time of the algorithm, especially when large datasets and complex operations are involved [31]. Efficient algorithms created through time complexity analysis use processor and memory resources more effectively. This is especially important in large-scale systems. In the time complexity analysis applied to the developed Hybrid Model-1 and Hybrid Model-2 algorithms, each step of the algorithms was examined, and their time complexities were determined. The most time-consuming step was determined. The technical details of the analysis in the study were taken from the reference [32].

Since the algorithms of the hybrid models have similar approaches, their time complexities are shown step by step in Table 1. In the notations in Table 1, the f represents the number of features considered in the algorithm. This is important in cases where the algorithm must process each feature separately. The n represents the number of samples in the dataset. It represents the total amount of data processed by the algorithm. The d represents the size of each data sample or the number of features. It shows how many features each sample in the dataset consists of. The k represents the number of clusters or the parameter used in clustering algorithms. It is usually used in the calculation of clustering metrics.

In the time complexity analysis performed, the overall time complexity of both hybrid models was found to be O(f × (n^2^ + k × n × d). According to this result, as the number of features increases, the total processing time increases proportionally. The size of the data affects the processing time quadratically. As the data size, number of data, and number of clusters increase, calculations take longer, but they are not as effective as the size of the data.

### 2.4. Anomaly Detection Performance Evaluation

By considering anomaly detection as a clustering, the silhouette score, Davies–Bouldin index and Calinski–Harabasz index [33,34] were used in the performance evaluation of the hybrid models. The silhouette score [35,36] is a metric used to evaluate the quality of clustering in data. It measures how well a data point fits the cluster it belongs to. It takes values between −1 and 1. It is considered that silhouette scores between 0.25 and 0.5 are acceptable, and 0.5 and above is a good result [37,38]. Equation (7) states the silhouette score calculation:

(7) $$s_{i}=\frac{b\left(i\right)-a(i)}{max(a\left(i\right),b\left(i\right))}$$

In Equation (7), a(i) is the average distance of the data point to the other data points in its cluster, and b(i) is the average distance of the data point to the nearest neighbor cluster.

The Davies–Bouldin index [35] is a metric used to evaluate the performance of clustering algorithms. This index is based on the idea that clusters should be tight and well separated. It ranges from 0 to infinity, with lower values indicating a better clustering performance. Equation (8) states the Davies–Bouldin index (DB) calculation:

(8) $$DB=\frac{1}{k}\sum_{i=1}^{k}{max}_{j\neq i}\left(\frac{\sigma_{i}+\sigma_{j}}{d\left(c_{i},c_{j}\right)}\right)$$

In Equation (8), k is the number of clusters, σ_i_ is the average distance within the cluster i, and d(c_i_,c_j_) is the distance between cluster centers c_i_ and c_j_.

The Calinski–Harabasz index [39,40] is another metric used to evaluate the quality of clustering algorithms. This index is based on the idea that good clusters should have high inter-cluster variance and low within-cluster variance. It does not have a fixed range, but higher values are preferred and can vary significantly depending on the dataset and number of clusters. Equation (9) states the Calinski–Harabasz index (CH) calculation:

(9) $$CH=\frac{tr(B_{k})/(k-1)}{tr(W_{k})/(n-k)}$$

In Equation (9), tr(B_k_) refers to the total squares between clusters, tr(W_k_) refers to the total squares within clusters, k is the number of clusters, and n is the total data.

Besides detecting anomalies in driving data, unnecessary consumption amounts caused by the detected anomalies were also detected. Since the anomalies in the driving data represent extraordinary situations, it was assumed that they would negatively affect the vehicle energy consumption, and the sum of the anomalies in the power values revealed unnecessary energy consumption. Abnormal energy consumption was calculated by including the appropriate time unit in the power values labeled as anomalies in the instantaneous power values obtained from the CAN bus records. The total consumed energy (E) calculation is explained in Equation (10):

(10) $$E=\int_{i}^{j}P_{i}*\frac{t_{i}}{3600}$$

In Equation (10), i and j represent the detected anomaly series starting and ending sequence numbers. P_i_ is the abnormal instantaneous power value in kilowatts. t_i_ is the instantaneous time change in seconds. As a result, the energy consumption value determined as an anomaly is obtained in kilowatt-hours.

### 2.5. Chaoticity Assessment

Chaoticity analysis serves as a vital tool in gaining a deeper understanding of the dynamics within driving data, particularly in assessing predictability and identifying potentially anomalous behaviors. This study utilized chaoticity metrics such as the Lyapunov exponent, Kolmogorov–Sinai entropy, and fractal dimensions to discern the systematic or chaotic nature of driving patterns. These metrics can indicate instability or unexpected shifts in driving conditions, which are crucial for guiding the design of vehicle safety and control systems. By enhancing anomaly detection and sensitivity, chaoticity analysis plays a significant role in improving driving safety. For instance, the identification of sudden chaotic behavior may be linked to conditions in which a driver could lose control, thereby providing critical input for early warning systems that predict and alert drivers to potential accident risks. Additionally, the inherently unpredictable nature of chaotic driving behavior suggests that minor changes can lead to substantial differences over time, making it difficult to foresee unexpected events. Chaoticity analysis offers a framework for developing risk management strategies by allowing for the examination of such unpredictability. For example, in high-risk scenarios like driving on slippery roads, vehicle control algorithms can be designed to minimize chaotic behavior. Erratic driving patterns may also indicate underlying factors that compromise driving safety, such as driver fatigue or distraction. By detecting moments of chaotic behavior, vehicle control systems can generate appropriate interventions. Moreover, chaoticity analysis can identify critical safety concerns based on traffic density or road conditions, thereby aiding in the optimization of traffic management strategies.

Chaoticity assessment was conducted with the methods preferred in the literature. The Maximal Lyapunov Exponent [23] is one of the most widely used metrics in analyzing chaotic systems and is considered a standard method in time series analysis. The negative value of the lambda value calculated in this method indicates that the system is dynamically stable, the zero value indicates that the system is orderly and neutral, and the positive value indicates that a chaotic system exists. The basic calculation for the Lyapunov Exponent is given in Equation (11):

(11) $$\lambda =\frac{1}{k\tau }\sum_{j=1}^{k}\log\left(||Y\left(i\right)-Y({nn}_{j})||\right)$$

In Equation (11), λ is the Lyapunov exponent, τ is the delay time, k is the number of nearest neighbors, nn_j_ is the j of Y(i). It represents the nearest neighbor.

The Kolmogorov–Sinai entropy [25,41] quantifies the unpredictability and complexity of the system. Higher entropy values indicate more complex and unpredictable behavior. Metric Entropy measures the rate at which the system produces information and is used in the context of information theory. It is used as a suitable metric to evaluate the complexity and chaotic properties of time series data. A situation where the Kolmogorov–Sinai entropy value is infinite indicates that the time series being processed is random, a situation where it is zero indicates that it is regular or semi-regular, and a value between zero and infinity indicates a chaotic time series. The basic steps used in the Kolmogorov–Sinai entropy calculation include phase space reconstruction, distance matrix calculation, counting distances below the threshold, and entropy calculation.

The steps used in Kolmogorov–Sinai entropy calculation include phase space reconstruction, probability distribution, Shannon entropy calculation, and Kolmogorov–Sinai entropy calculation. The phase space reconstruction, also known as Takens’s theorem, is shown in Equation (12). In Equation (12), Y(i) is a point in the reconstructed phase space, x(i) is the data point in the time series, τ is the delay time, and m is the embedding dimension:

(12) $$Y\left(i\right)=[x\left(i\right),x\left(i+\tau \right),x\left(i+2\tau \right),\ldots ,x\left(i+\left(m-1\right)\tau \right)]$$

Determining the probability distribution involves calculating it using the distances between points in the reconstructed phase space. Distances are usually calculated by the Euclidean distance. The formula used to determine the probability distribution is shown in Equation (13):

(13) $$d_{ij}=\sqrt{\sum_{k=1}^{m}{\left(Y_{i}\left(k\right)-Y_{j}\left(k\right)\right)}^{2}}$$

In this equation (13), d_ij_ represents the linear distance between two points in the phase space. Y_i_ and Y_j_ are the points in the phase space, and Y_i_(k) represents the kth component of the Y_i_ point in the phase space.

The probability distribution is created using the distances in the Shannon entropy calculation. The Shannon entropy calculation is given in Equation (14):

(14) $$H\left(p\right)=-\sum_{i=1}^{N}p_{i}\log\left(p_{i}\right)$$

In Equation (14), H(p) represents the Shannon entropy, p_i_ represents the probability of each state in the probability distribution, and N represents the number of states in the probability distribution.

In the Kolmogorov–Sinai entropy calculation, Shannon entropy is used and the time average is taken into account. The formula for the relevant calculation is given in Equation (15):

(15) $$K_{s}=\frac{H(p)}{\tau }$$

The fractal dimension [42,43,44] is used to understand the complexity and self-similarity of data models. The box-counting dimension calculates the fractal dimension by dividing the dataset into boxes. It is the most common, easy to calculate, and widely accepted method in time series analysis. This method is widely used to identify fractal properties of data and is ideal for measuring complexity in time series data. If the fractal dimension result is less than 1, the structure considered is in one dimension and shows straight-line behavior; if it is between 1 and 2, it means that it is two-dimensional, like a curve or surface, but does not fill a completely two-dimensional area; if it is between 2 and 3, it looks like a surface but has three dimensions. It does not fill a three-dimensional volume, and the fact that it is greater than 3 indicates that a structure that is complex enough to cover a four-dimensional space has been formed. When calculating the fractal dimension with the box-counting method, a grid of different scales (from small to large) is applied to the fractal shape under examination. At each grid scale, the filled boxes on the fractal shape are counted. The numbers of filled boxes corresponding to different grid scales are plotted on the log-log plot. The slope of the log-log plot gives the fractal dimension. The fundamental formula required for box-counting dimension calculation, the sub-method used in fractal dimensioning, is given in Equation (16). In Equation (16), D is the fractal dimension, ε is the box dimension of the grid, and N is the full box number:

(16) $$D=\underset{\varepsilon \to 0}{\lim}\frac{\log N(\varepsilon )}{\log\frac{1}{\varepsilon }}=-\underset{\varepsilon \to 0}{\lim}\frac{\log N(\varepsilon )}{\log\varepsilon }$$

## 3. Results

For anomaly detection on driving data, the performance evaluation of Hybrid Model-1 and Hybrid Model-2, which were created with the combination of the LSTM, LOF, and Mahalanobis distance sub-models, on two different driving datasets was carried out. The training and reconstruction processes of the LSTM-Autoencoder sub-models were completed and performance graphs were shared. Then, the anomaly rates of the LSTM-autoencoder, LOF, and Mahalanobis sub-models were shared. The Hybrid Model-1 and Hybrid Model-2 anomaly rates were compared on the same data. In the last part, the results of the silhouette score, Davies–Bouldin index, and Calinski–Harabasz index, which are performance evaluation criteria according to two different datasets, are shown. Chaoticity assessment of the driving data was also carried out. The Lyapunov exponent, Kolmogorov–Sinai entropy, and fractal dimension metrics were used in the chaoticity assessment. The obtained results were evaluated together with the anomaly and chaoticity results.

### 3.1. Performance Results on Driving-A

**Figure 6:**
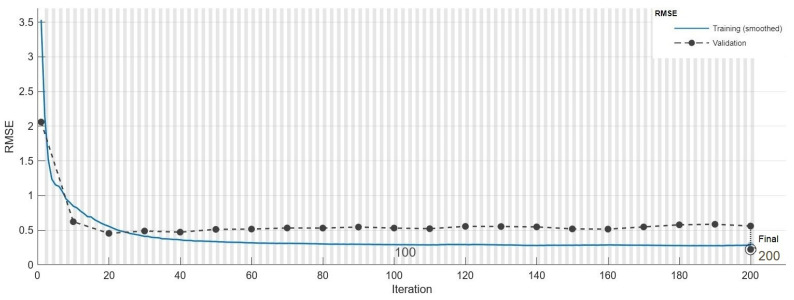
LSTM-Autoencoder training performance curve on Driving-A.

The LSTM-Autoencoder model was trained with two driving datasets. The RMSE value at the end of the training process carried out with Driving-A data was 0.211. The performance graph of the training process can be seen in Figure 6. When the graph was examined, it was thought that the error value did not change after approximately 150 epochs, and that the model training was completed at an acceptable level.

**Figure 7:**
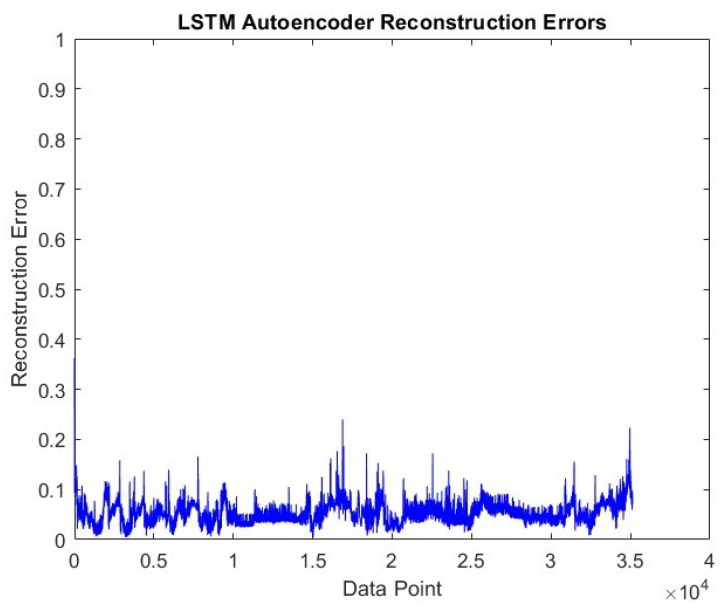
LSTM-Autoencoder reconstruction error graph on Driving-A.

The visual of the error values that emerged at the end of the post-training reconstruction process of the LSTM-Autoencoder model is shared in Figure 7. When the overall data are evaluated, the average error rate is around 0.06.

The rates resulting from the anomaly detection of the sub-models on the Driving-A data are shared in Table 2. Anomaly detections obtained with the LSTM, LOF, and Mahalanobis sub-models are divided according to the data characteristics, and the total anomaly rates detected by the sub-models are shared at the bottom. According to the total anomaly rates, the LSTM-Autoencoder and Mahalanobis distance showed approximately similar performances in the Driving-A data. Accordingly, the LOF has a lower anomaly rate. It has the lowest anomaly power among the data features. In contrast, pedal and speed had the highest anomaly rates.

Table 3 presents the anomaly detection rates for Driving-A data using Hybrid Model-1 and Hybrid Model-2, which combine LSTM-Autoencoder with the LOF and Mahalanobis distance methods. When comparing the total anomaly detection rates, Hybrid Model-1 outperformed its sub-methods, achieving 5.27% and 12.07% higher detection rates compared to the LSTM-Autoencoder and LOF, respectively. Similarly, Hybrid Model-2 demonstrated a 12.62% and 11.47% improvement over the LSTM-Autoencoder and Mahalanobis distance, respectively. These results confirm that the hybrid models met the expected performance increase in anomaly detection. Additionally, Hybrid Model-2 was observed to detect a higher percentage of anomalies in Driving-A data compared to Hybrid Model-1, further validating the effectiveness of the hybrid approach.

The performance evaluation results of the anomaly detection performed by Hybrid Model-1 on the Driving-A data are in Table 4. As a result of the silhouette score evaluation among the preferred performance metrics in the study, it was seen that although there were features with low scores, there were features with sufficient and high rates. In the Davies–Bouldin index results, speed showed the best value, followed by pedal data. In the Calinski–Harabasz index, speed and power data were the features that yielded the best results.

As a result of the calculation made according to Equation (16) in Driving-A data, 1.7055 kWh was obtained. This value expresses the total amount of energy consumed on the drive. However, according to the value obtained as a result of the sum of the anomaly values detected in the power feature of the Hybrid Model-1’s Driving-A data, the potential energy saving was 0.6342 kWh. According to this value, it has been shown that there can be a 37.18% potential energy saving if Driving-A is carried out without any anomalies.

The performance evaluation results of the anomaly detection performed with Hybrid Model-2 on Driving-A data are shared in Table 5. The silhouette scores were higher than those of Hybrid Model-1. According to the features, the silhouette scores were evaluated as having sufficient rates. In the Davies–Bouldin index results, speed and pedal showed the best values. As for the Calinski–Harabasz index, speed and pedal data were the features that gave the best results.

According to the anomaly values detected by Hybrid Model-2 in the Driving-A data, where the total energy consumption was 1.7055 kWh, the potential energy saving was found to be 0.3215 kWh. In this regard, 18.85% of unnecessary energy consumption was made for this drive.

**Figure 8:**
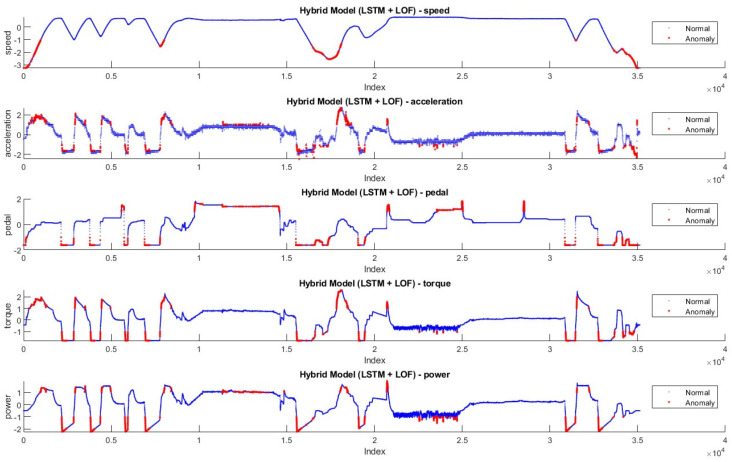
Anomalies detected by Hybrid Model-1 in Driving-A.

**Figure 9:**
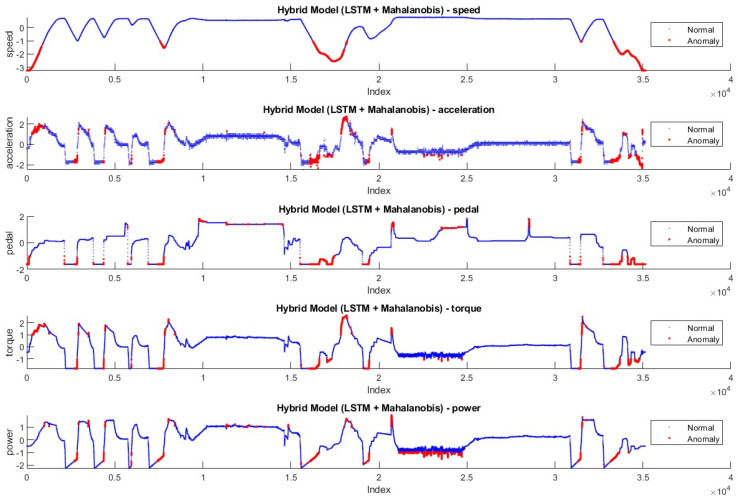
Anomalies detected by Hybrid Model-2 in Driving-A.

In Figure 8 and Figure 9, the anomaly distributions in Driving-A with Hybrid Model-1 and Hybrid Model-2 are shown. According to the data features, anomaly values are shown in red dots, and normal values are shown in blue. Based on the anomaly threshold approaches given in the literature [45,46,47], anomalies that disrupt the flow and generally have rapid changes in data features are labeled as anomalies.

### 3.2. Performance Results for Driving-B

**Figure 10:**
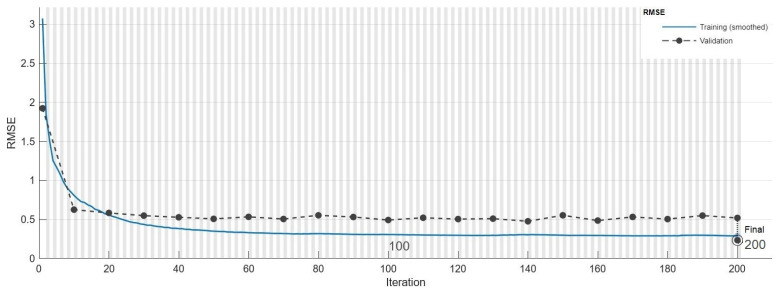
LSTM-Autoencoder training performance curve on Driving-B.

The RMSE value of the LSTM-Autoencoder sub-model with Driving-B data after 200 epochs of training was 0.2095. In terms of the learning process, the LSTM-Autoencoder sub-model showed a similar performance for both driving datasets. The RMSE values of the training process carried out with Driving-B data are seen in Figure 10. Similar to the training process carried out with the first dataset, it was observed that the error value stabilized after approximately 150 epochs in this training process, and it was considered that the model training was completed sufficiently.

**Figure 11:**
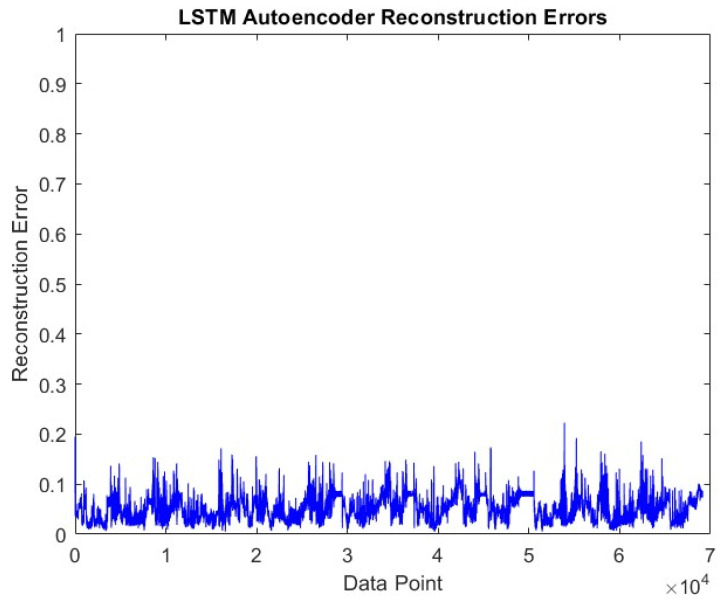
LSTM-Autoencoder reconstruction error graph on Driving-B.

The reconstruction error shown by the LSTM-Autoencoder sub-model in the Driving-B data is shared in Figure 11. The average reconstruction error in these data is higher than in the Driving-A data and is around 0.09.

The anomaly rates detected in the sub-models in the Driving-B data are shared in Table 6. According to the total anomaly rates in Table 6 and Table 7, LSTM-Autoencoder was able to detect 16.62%, the LOF was able to detect 8.58%, and the Mahalanobis distance was able to detect 7.80%. Hybrid Model-1, developed with traditional methods, showed an anomaly detection performance of 21.89%, and Hybrid Model-2 showed 20.38%. According to these results, the anomaly detection performance of the hybrid models is superior to that of traditional methods. Among the data features, power data showed the lowest anomaly rate in the LSTM-Autoencoder and LOF sub-models, while acceleration had the lowest anomaly rate in the Mahalanobis sub-model. In contrast, speed and pedal data generally had high anomaly rates.

The anomaly rates realized for Driving-B data by Hybrid Model-1 and Hybrid Model-2 are shared in Table 7. When the total anomaly rates are compared, Hybrid Model-1 was able to detect anomalies more successfully than its sub-methods LSTM-Autoencoder and LOF by 5.27% and 13.31%, respectively. Hybrid Model-2 was superior to its sub-methods LSTM-Autoencoder and the Mahalanobis distance by 3.76% and 12.58%, respectively. The anomaly detection performance increases of the hybrid models largely provided the expected values. When the hybrid models were compared among themselves, Hybrid Model-1 was able to detect anomalies at a higher rate.

Table 7 details the anomaly detection performance for Driving-B data. Hybrid Model-1 detected anomalies 5.27% and 13.31% more effectively than its LSTM-Autoencoder and LOF sub-methods, respectively, while Hybrid Model-2 outperformed the LSTM-Autoencoder and Mahalanobis distance by 3.76% and 12.58%, respectively. The performance increases achieved by the hybrid models largely align with the anticipated values. When comparing the hybrid models, Hybrid Model-1 demonstrated a superior anomaly detection rate.

The performance evaluation results of the abnormal detection performed with Hybrid Model-1 on Driving-B data are shared in Table 8. In the silhouette score comparison, it was seen that all the features except the pedal had sufficient performance. Speed data showed the best value in the Davies–Bouldin index results. As for the Calinski–Harabasz index, speed and power data were the features that gave the best results.

As a result of including the data in the power feature of the Driving-B dataset according to Equation (16), the total energy consumption was seen as 0.1129 kWh. The potential energy saving as a result of the sum of the anomalies detected in the power feature of the Driving-B data of Hybrid Model-1 was 0.0286 kWh. According to this value, it has been shown that Driving-B can provide a potential energy saving of 25.33% without any anomalies.

The anomaly detection performance evaluation results of Hybrid Model-2 in Driving-B are shared in Table 9. The silhouette scores were evaluated as sufficient in all features. Speed and power data showed the best values in the Davies–Bouldin index results. Similarly, in the Calinski–Harabasz index, speed and power data gave the best results.

As a result of the anomaly detection made by Hybrid Model-2 in the Driving-B data, where the total energy consumption was 0.1129 kWh, the potential energy saving was found to be 0.0498 kWh. According to this value, 44.11% of unnecessary energy was consumed while driving.

**Figure 12:**
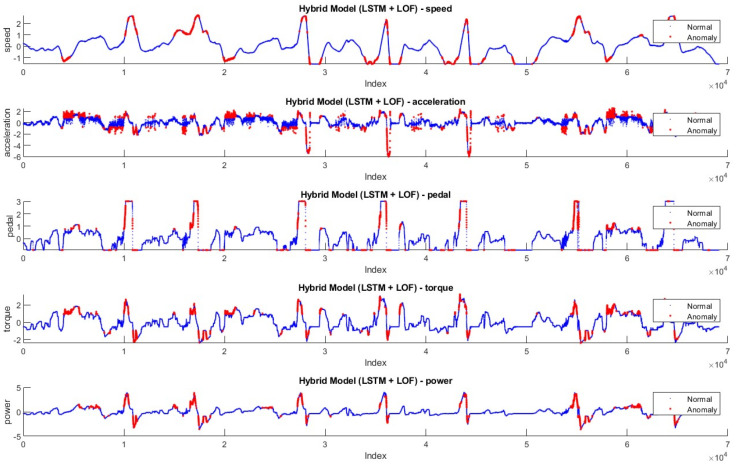
Anomalies detected by Hybrid Model-1 on Driving-B.

**Figure 13:**
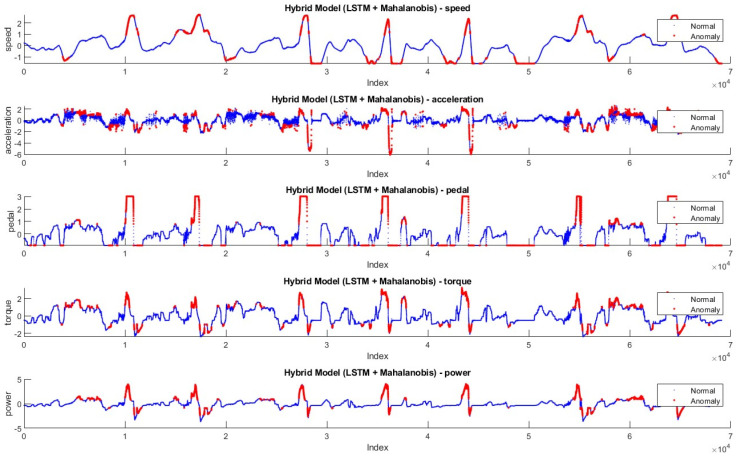
Anomalies detected by Hybrid Model-2 on Driving-B.

Figure 12 and Figure 13 show the anomaly distribution in the Driving-B data according to Hybrid Model-1 and Hybrid Model-2. Similar to the threshold approaches preferred in the Driving-A data, the anomaly values are in red, and the normal values are blue dots. Since this drive represents city driving with frequent stop-and-go, the data characteristics show more fluctuation. Sudden changes throughout the anomaly detection are labeled as anomalies.

**Figure 14:**
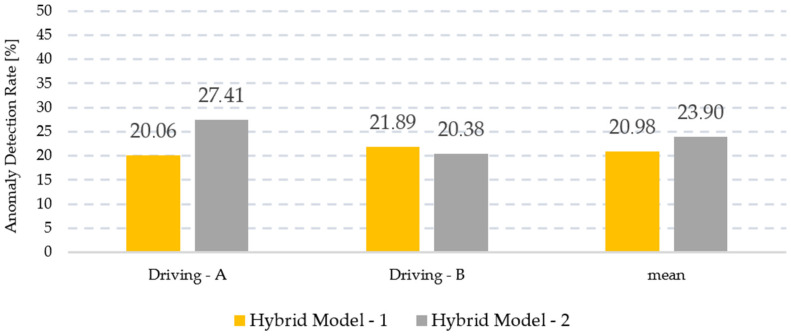
Anomaly detection performances of hybrid models.

**Figure 15:**
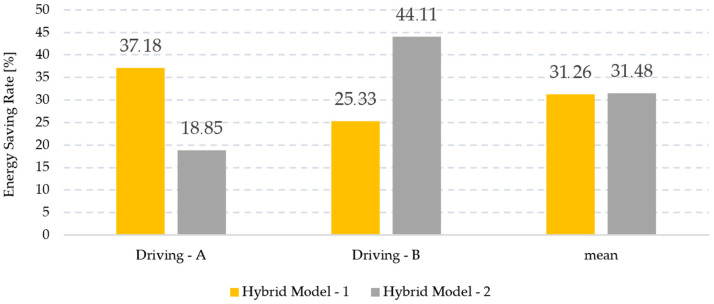
Energy-saving performances of hybrid models.

Figure 14 and Figure 15 provide a general visualization of the study results. In Figure 14, the anomaly detection performances of the hybrid models in the driving data are shared with their average rates. In Figure 15, the excessive energy consumption rates caused by the anomalies detected by the hybrid models are presented with their average values. Looking at the average ratio columns, it is seen that the energy-saving performance of Hybrid Model-2 is similar to that of Hybrid Model-1, but when the same evaluation is made for the anomaly detection performance, Hybrid Model-2 has a higher performance. Since this study focused on anomaly detection and there was no significant difference in energy savings, it was decided that Hybrid Model-2 was superior.

The energy consumption associated with the algorithms during anomaly detection has a notable impact when compared to the overall power consumption of the ride. Specifically, the potential energy savings identified through anomaly detection highlight the significant amount of excess energy that would be consumed if such anomalies were not detected. This finding is particularly relevant for energy management and driving range optimization. This study demonstrates that, on average, approximately 32% of unnecessary energy is consumed during drives without anomaly detection. While this energy saving may appear minimal when expressed in kilowatt-hours, the broader implications are substantial, particularly when considering an entire electric vehicle fleet or a mobility ecosystem characterized by high energy consumption. The material and environmental benefits of these savings become even more pronounced when factoring in future developments, such as the increasing impact of carbon taxes. By presenting a method capable of addressing such issues effectively, the significance of the result is further amplified.

### 3.3. Chaoticity Assessment Results

A chaoticity assessment of the Driving-A and Driving-B real-world driving datasets was carried out with the help of appropriate measurements. The chaoticity assessment was carried out with the vehicle speed data in the dataset due to the reduction in the processing intensity and its good representation of the dataset. During the chaoticity assessment phase, Lyapunov exponent, Kolmogorov–Sinai entropy, and fractal dimension calculations, which are frequently used methods in the literature, were made. According to the results obtained, it was seen that the chaoticity in both driving datasets was low.

**Figure 16:**
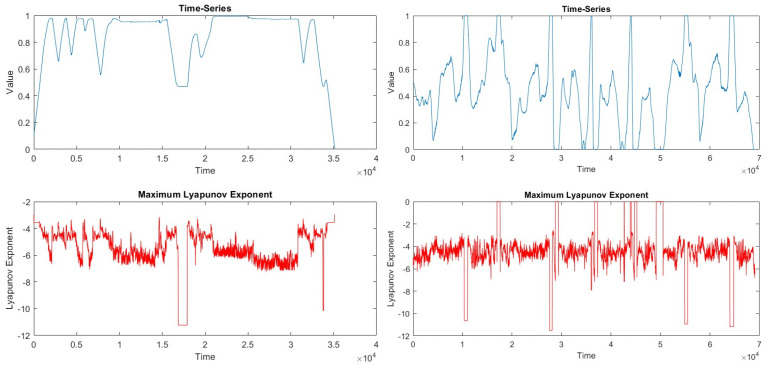
Lyapunov exponentials: Driving-A data (**left**) and Driving-B data (**right**).

**Figure 17:**
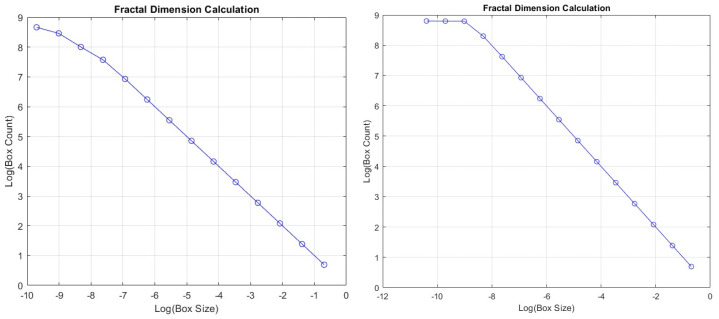
Fractal dimensions: Driving-A (**left**) and Driving-B (**right**).

In the assessment performed with the Driving-A driving dataset, the Lyapunov exponent was found to be −0.4439, the Kolmogorov–Sinai entropy was 0.0261, and the fractal dimension was 0.9278. The negative Lyapunov exponent value indicates that the system is stable and not chaotic, the Kolmogorov–Sinai entropy value is close to zero, the system is quite orderly and predictable, and the fractal dimension value is below 1, indicating that the system shows one-dimensional, that is, linear, behavior. In the assessment performed with the Driving-A driving dataset, the Lyapunov exponent was found to be −0.4439, the Kolmogorov–Sinai entropy was 0.0261, and the fractal dimension was 0.9278. When the metrics are evaluated together, it can be concluded that the system is generally orderly and predictable. A negative Lyapunov exponent indicates that the system is not chaotic, a low Kolmogorov–Sinai entropy indicates that the system is orderly, and a fractal dimension close to 1 indicates that the system exhibits a linear and orderly structure. Together, these results show that the data analyzed are not complex and chaotic but rather have an orderly and predictable structure. Figure 16 shows normalized vehicle speed data and Lyapunov exponent plots in Driving-A data. Figure 17 presents the logarithmic graph containing the box count and box size data created for the fractal dimension calculation.

The Driving-B data were similar to the results of the Driving-A data in the chaoticity assessment. The Lyapunov exponent was −0.4457, the Kolmogorov–Sinai entropy was 0.2216, and the fractal dimension was found to be 0.9084. According to these results, the Lyapunov value shows that the system is not chaotic and has a more stable structure. The Kolmogorov–Sinai entropy indicates that the system has a low level of complexity, which is consistent with the Lyapunov exponent, indicating that the system is not chaotic and is more stable. The fractal dimension value indicates that the system has a low level of complexity, which indicates that it is geometrically quite simple. Normalized vehicle speed data and Lyapunov exponent graphs of Driving-B data are seen in Figure 16. The logarithmic plot of the box number and box size data for the fractal dimension calculation is in Figure 17.

## 4. Discussion

Anomaly detection in mobility has the potential to provide more output than expected in transportation systems. Vehicle driving data provide the necessary material for techniques that are open to development, applicable, and can support various fields. In this study, the anomaly detection performances of two different hybrid models developed with the LSTM-Autoencoder, LOF, and Mahalanobis distance sub-models were compared on real driving data. The results show that the anomaly detection performance of the developed hybrid models is superior to that of traditional methods. In addition, a close relationship between anomaly and chaoticity was shown in the chaotic click analysis. Thanks to the detection of excessive energy consumption caused by anomalies, a longer driving range was provided.

The LSTM method used in Qin et al.’s study [5] was also used in this study. In addition, the anomaly detection performance has been supported by other techniques in the literature. In addition, detecting excessive energy consumption due to abnormalities also provides support for the driving range issue, which is of serious importance for electric vehicles. It is suggested that it would be more appropriate to include various traffic scenarios in future studies in Moso et al.’s study [21]. This study also evaluated the performances of anomaly detection models by taking into account multiple driving scenarios from the same perspective. It was shown that the developed hybrid models can process various data independent of the scenario. Yun et al. [22] used additional environmental components for detailed data collection. Anomaly detection of the drives was made based on data obtained from external sources rather than the internal data of the vehicle. Among the machine learning techniques, the One-Class-SVM, K-Means, HDBSCAN, and Minisom algorithms were used. Well-known techniques were used in the model learning phase and were successful. At the same time, working with real-world data increases the effectiveness of its work. In this study, performance increase was achieved by supporting LSTM-Autoencoder, a well-known machine learning method, with statistical methods. Additionally, in this study, the applicability and general validity of the model were demonstrated using real-world driving data. The effective methods used in Wickramasinghe et al.’s study [6] led to the development of a successful model. The perspective of the study covers the issue of safety in electric vehicles. The anomaly data obtained can successfully detect possible attacks and keep vehicle security at the highest level. In the study, Kia Soul and Hyundai YF Sonata vehicle data were used by the Hacking and Countermeasures Research Lab (HCRL). In the study, in addition to machine learning, there was a method used in common with the work of Wickramasinghe et al. While the commonly used method can be successful in terms of security, in this study, it was also successful in energy consumption optimization. In addition, the real-world driving data characteristics used in this study and the conditions under which the data were obtained are clearly shown. Barbosa et al. [23] achieved successful results with the Lyapunov exponent, which he preferred in his work. It has been shown that it can be worked on various datasets with the successful results it can provide in nonlinear systems. In this study, the results were obtained by using a similar method in time series. The preferred similar method was also successful at evaluating the chaotic state of vehicle driving data. In addition, this study includes Kolmogorov–Sinai entropy and fractal dimension results, making the chaoticity analysis comprehensive. Vogl [24] observed significant fluctuations in the Maximum Lyapunov Exponent and entropy in periods close to crisis. Similarly, a local increase was observed in the methods used in this study, and an increase in chaos was observed in these regions.

## 5. Conclusions

Anomaly detection methods from driving data are easily applicable, open to development, and promising techniques for different applications. In this study, two different driving data anomaly detections were made with two different hybrid models created by the combination of the LSTM-Autoencoder, LOF, and Mahalanobis distance sub-methods. The hybrid models showed a superior performance compared to that in traditional methods in anomaly detection. Thanks to the developed approach, anomaly detection can be performed comprehensively with CAN bus data without the need for additional sensor data. The important findings obtained in terms of energy efficiency also have the potential to easily determine the ideal energy requirement for a drive. In the future, predictive driving support systems can be developed by detecting chaotic behaviors.

The silhouette score, which is used in many studies for performance evaluation purposes, was carried out with the results of the Davies–Bouldin index and the Calinski–Harabasz index. In addition to anomaly detection performances, unnecessary energy consumption values caused by detected anomalies were also determined. In this way, the potential energy saving amounts in driving were determined. Real driving data were also assessed in terms of chaoticity, and infrastructure was created to predict the future state of the driving style and was associated with the anomaly results.

The inferences made from the results obtained in the research include that the anomaly detection performances of hybrid models are better than the LSTM, LOF, and Mahalanobis distance methods. Both hybrid models can generalize independently of the data. The LOF sub-model has lower anomaly rates than the Mahalanobis distance sub-model but has the potential to improve the overall performance when used in hybrid models. According to the Davies–Bouldin index and Calinski–Harabasz index, speed and power data stand out as the best-performing features overall. On the Driving-A dataset, Hybrid Model-1 created greater energy saving potential, while Hybrid Model-2 had a higher anomaly detection performance. In the Driving-B dataset, Hybrid Model-1 was more successful in terms of anomaly detection, while Hybrid Model-2 offered higher energy-saving potential. In light of these evaluations, it can be seen that Hybrid Model-2, which consists of the combination of the LSTM-Autoencoder and Mahalanobis distance, is more effective at detecting anomalies and better captures the energy-saving potential. The fact that speed and pedal features have higher anomaly rates in different datasets shows that these features need to be analyzed more carefully.

The chaoticity analysis revealed that there was no significant chaotic behavior in the Driving-A and Driving-B datasets, indicating that both rides were stable and exhibited low complexity. This suggests that the driver behaviors in these scenarios did not present a potential accident risk, and the driving characteristics remained within predictable bounds. The two driving behaviors were comparable in terms of chaoticity. However, moments of relatively higher chaoticity were observed, which aligned closely with instances of anomaly detection. This finding underscores the relationship between anomaly and chaoticity, suggesting that a ride with a high incidence of anomalies may also exhibit elevated chaotic content.

This study, which makes valuable contributions to the literature, also contains deficiencies that can guide future studies. Future studies on this subject will focus on improving the anomaly detection performance of hybrid models, detecting drivers from the density of anomalies in driving data, determining road quality, performance evaluations of different hybrid models, separating anomalies according to their types, detecting anomalies in synthetic driving data, and detailing the anomaly-chaoticity relationship.

## Figures and Tables

**Table 1:** Time complexity analysis of hybrid models.

Algorithm Step	Time Complexity O Notation
Steps 1–2	O(1)
Step 3	O(n × d)
Step 4	O(n)
Steps 5–12	O(f × (n^2^ + k × n × d)
Steps 13–14	O(n)
Steps 15–17	O(n)
Steps 18–20	O(f × n)

**Table 2:** Anomaly rates of sub-models on Driving-A.

Data Feature	LSTM	LOF	Mahalanobis Distance
Speed	6.06%	3.75%	13.22%
Acceleration	6.87%	2.85%	9.54%
Pedal	9.57%	3.84%	11.48%
Torque	6.65%	2.65%	9.16%
Power	5.48%	1.78%	5.29%
TOTAL	14.79%	7.99%	15.94%

**Table 3:** Anomaly rates of hybrid models on Driving-A.

Data Feature	Hybrid Model-1	Hybrid Model-2
Speed	8.15%	13.46%
Acceleration	8.44%	17.06%
Pedal	11.68%	17.73%
Torque	8.10%	16.55%
Power	6.57%	13.19%
TOTAL	20.06%	27.41%

**Table 4:** Performance results for Driving-A with Hybrid Model-1.

Data Feature	Silhouette	Davies–Bouldin Index	Calinski–Harabasz Index
Speed	0.74	0.44	12,251.48
Acceleration	0.43	1.85	6840.92
Pedal	0.47	0.82	6908.50
Torque	0.48	1.76	7625.86
Power	0.37	1.17	12,723.62

**Table 5:** Performance results for Driving-A with Hybrid Model-2.

Data Feature	Silhouette	Davies–Bouldin Index	Calinski–Harabasz Index
Speed	0.87	0.35	91,618.38
Acceleration	0.53	1.94	5134.52
Pedal	0.67	0.35	37,947.00
Torque	0.56	1.70	6282.94
Power	0.55	0.99	11,263.14

**Table 6:** Anomaly rates of sub-models in Driving-B.

Data Feature	LSTM	LOF	Mahalanobis Distance
Speed	5.80%	4.67%	7.80%
Acceleration	5.80%	3.95%	4.54%
Pedal	5.96%	3.81%	8.24%
Torque	5.41%	3.36%	4.96%
Power	4.53%	2.53%	6.17%
TOTAL	16.62%	8.58%	7.80%

**Table 7:** Anomaly rates of hybrid models on Driving-B.

Data Feature	Hybrid Model-1	Hybrid Model-2
Speed	8.34%	12.05%
Acceleration	7.79%	12.92%
Pedal	7.79%	18.28%
Torque	7.18%	10.78%
Power	5.98%	10.35%
TOTAL	21.89%	20.38%

**Table 8:** Performance results for Driving-B with Hybrid Model-1.

Data Feature	Silhouette	Davies–Bouldin Index	Calinski–Harabasz Index
Speed	0.52	2.40	3707.32
Acceleration	0.66	3.36	104.76
Pedal	0.35	19.76	76.70
Torque	0.56	13.85	2362.25
Power	0.67	3.80	3482.46

**Table 9:** Performance results in Driving-B with Hybrid Model-2.

Data Feature	Silhouette	Davies–Bouldin Index	Calinski–Harabasz Index
Speed	0.60	1.85	10,139.20
Acceleration	0.62	4.89	1545.93
Pedal	0.59	2.89	6971.01
Torque	0.56	4.34	2120.08
Power	0.73	1.83	11,795.85

## Data Availability

Data is available and can be provided upon request.
